# Hyperbaric oxygenation alters temporal expression pattern of superoxide dismutase 2 after cortical stab injury in rats

**DOI:** 10.3325/cmj.2012.53.586

**Published:** 2012-12

**Authors:** Ana B. Parabucki, Iva D. Božić, Ivana M. Bjelobaba, Irena C. Lavrnja, Predrag D. Brkić, Tomislav S. Jovanović, Danijela Z. Savić, Mirjana B. Stojiljković, Sanja M. Peković

**Affiliations:** 1 Department of Neurobiology, Institute for Biological Research “Siniša Stanković,” University of Belgrade, Serbia; 2 Institute of Medical Physiology “Richard Burian,” School of Medicine, University of Belgrade, Belgrade, Serbia; 3 Centre for Hyperbaric Medicine, Belgrade, Serbia

## Abstract

**Aim:**

To evaluate the effect of hyperbaric oxygen therapy (HBOT) on superoxide dismutase 2 (SOD2) expression pattern after the cortical stab injury (CSI).

**Methods:**

CSI was performed on 88 male Wistar rats, divided into control, sham, lesioned, and HBO groups. HBOT protocol was the following: pressure applied was 2.5 absolute atmospheres, for 60 minutes, once a day for consecutive 3 or 10 days.‎ The pattern of SOD2 expression and cellular localization was analyzed using real-time polymerase chain reaction, Western blot, and double-label fluorescence immunohistochemistry. Neurons undergoing degeneration were visualized with Fluoro-Jade^®^B.

**Results:**

CSI induced significant transient increase in SOD2 protein levels at day 3 post injury, which was followed by a reduction toward control levels at post-injury day 10. At the same time points, mRNA levels for SOD2 in the injured cortex were down-regulated. Exposure to HBO for 3 days considerably down-regulated SOD2 protein levels in the injured cortex, while after 10 days of HBOT an up-regulation of SOD2 was observed. HBOT significantly increased mRNA levels for SOD2 at both time points compared to the corresponding L group, but they were still lower than in controls. Double immunofluorescence staining revealed that 3 days after CSI, up-regulation of SOD2 was mostly due to an increased expression in reactive astrocytes surrounding the lesion site. HBOT attenuated SOD2 expression both in neuronal and astroglial cells. Fluoro-Jade^®^B labeling showed that HBOT significantly decreased the number of degenerating neurons in the injured cortex.

**Conclusion:**

HBOT alters SOD2 protein and mRNA levels after brain injury in a time-dependent manner.

Traumatic brain injury (TBI) is among the most disabling injuries and represents the major health problem worldwide (1). TBI involves primary and secondary injury. Primary injury is the result of immediate mechanical damage of neural pathways that occurs at the time of injury and triggers a cascade of events known as secondary injury, which causes further damage to the brain. Secondary injury evolves over time and may persist for months to years after TBI causing primarily unaffected neurons to degenerate. Therefore, it should be considered as a chronic disease process and main target for potential therapies (2,3).

As a part of secondary post-injury cascade, oxidative stress, among the other events, is a prominent one (1). It has been demonstrated that reactive oxygen species (ROS), if exceed the capacity of the anti-oxidative defense, lead to oxidative stress and cellular damage after brain trauma (4-6). Additionally, it has been shown that depletion of antioxidant systems following trauma could adversely affect synaptic function and plasticity (7).

The first line of defense against ROS is in the place where it all begins – mitochondria. Manganese superoxide dismutase (SOD2), located in the mitochondrial inner membrane and matrix, is a critical antioxidant enzyme that catalyzes the dismutation of superoxide radical to oxygen and hydrogen peroxide. In different brain pathologies, the induction of SOD2 varies and depends on the type of injury (8), and most data point out the neuroprotective role of SOD2 in brain injury (9,10). In addition, experiments with SOD2 deficient mice who suffered from early degeneration have shown how essential this enzyme was for normal brain functioning (11,12). It has also been found that SOD2 expression was directly correlated with the grade of brain tumors in humans (13).

Despite the amount of work that has been done in order to better understand TBI, an adequate therapy is still lacking. In the past decade, hyperbaric oxygen therapy (HBOT) became one of the more frequently used medical tools and it appears to be a good therapeutic solution for a variety of conditions (14). Beneficial effects of HBOT as adjuvant therapy with surgery were also observed in the treatment of complex war injuries. Irrespectively of the type of surgical strategy applied, HBOT significantly reduced the frequency of wound complications and the time to wound stabilization (15). HBOT is a therapeutic approach where the patient is exposed to 100% oxygen at pressures higher than ambient (1 atmosphere absolute [1 ATA]). This leads to an increased blood oxygen level, which than can penetrate to ischemic areas more deeply than under normobaric conditions (16-18). In our recently published article (19), we have shown that HBOT can recover locomotor performances in rats after the brain injury. Although there is a large body evidence that HBOT is useful as a therapy for brain injury (18-23), there is also data indicating that the use of hyperbaric oxygen can have serious side effects (14,24-26). The main concern in HBOT is oxidative stress and/or oxygen toxicity that can affect multiple organs. ‎However, these unwelcome side-effects have often been dependent on treatment parameters – pressure and duration of the ‎treatment (25,27,28). The data on the exact mechanisms by which HBOT exerts its positive effects are deficient. Thus, in the present study we investigated the effect of HBOT on temporal expression pattern and cellular distribution of SOD2 after cortical stub injury (CSI).

## Methods

### Animals

The study used 88 ten weeks old male Wistar rats, weighing 250 ± 30 g at the time of surgery. Animals were housed 4 per cage and maintained at the animal facility of the Institute for Biological Research “Siniša ‎Stanković” (Belgrade, Serbia) in accordance with institutional guidelines, ‎under standard conditions (23 ± 2°C, 12h/12h light/dark cycle, food and water *ad libitum*). After the acclimatization period of one week, animals were randomly organized into the following groups (n = 8 per group): control group (C) – intact rats; control HBO group (CHBO) – intact rats subjected to the HBO protocol for 3 and 10 consecutive days; sham group (S) – animals that underwent surgical procedure without skull opening, sacrificed 3 or 10 days post injury (dpi); sham HBO group (SHBO) – the animals that underwent sham surgery and were subjected to the HBO protocol for 3 or 10 consecutive days; lesion group (L) – the animals that passed CSI and were sacrificed 3 or 10 dpi; and lesion HBO group (LHBO) – CSI rats subjected to the HBO protocol for 3 or 10 consecutive days. Experimental protocol received prior approval from the Ethical Committee of the School of Medicine, University of Belgrade (No. 3027/2), and was conducted in strict accordance with recommendations given in NIH Guide for the Care and Use of Laboratory Animals (NIH publication No. 85-23, *http://grants.nih.gov/grants/olaw/Guide-for-the-Care-and-Use-of-Laboratory-Animals.pdf*). All efforts were made to minimize the number of used animals and their suffering.

### Surgical procedure

A previously described model of cortical stab injury (CSI) was used (29). Before starting the surgical procedure, rats were anesthetized with ether. After the onset of anesthetic, rats were shaved and placed in the stereotaxic frame. Using a sterile scalpel blade the head was incised along the midline of the scalp to expose the bregma, and the bleeding was minimized with cotton swabs. Handheld 1-mm-wide dental drill was inserted vertically in the cranium on the left side. The coordinates of stab lesion to the left cortex were as follows: 2 mm posterior to the bregma, 2 mm lateral from the midline, and to a depth of 2 mm into the brain (30). The incision was closed with sutures. Rats from the sham control groups were anesthetized, placed in the stereotaxic frame, and subjected to the same surgical procedure, without causing further damage to the skull. Animals were placed in a heated room and monitored while recovering from anesthesia. Intact, age-matched animals were processed as controls. After the surgery, the rats were kept at warm and left up to 2 hours to recover before starting HBOT protocol.

### HBOT protocol

The rats were placed into experimental HBO chambers and exposed to 100% oxygen according to the following protocol: 10 minutes compression, 2.5 atmospheres absolute (ATA) for 60 minutes, and 10 minutes decompression. HBOT was performed once a day, for 3 or 10 consecutive days. This protocol corresponds to a standard hyperbaric oxygen treatment that is routinely used in the clinical setting of Center for Hyperbaric Medicine, Belgrade, Serbia (26), and is in line with the recommendations of The Committee of the Undersea and Hyperbaric Medical Society that a treatment pressure only from 2.4 to 3.0 ATA should be used as appropriate (31). Each exposure was started at the same hour to exclude any confounding issues associated with the changes in biological rhythm. Body temperature was not changed significantly after the HBOT.

### Tissue preparation

Five rats were taken from each group for immunoblot and gene expression analyses. After the end of treatment protocol (at 3 or 10 dpi) animals were sacrificed by decapitation under deep ether anesthesia. Immediately after decapitation, the brains were quickly removed from the skull. From injured (left) cortices 2 mm sections around the center of lesion were dissected on ice and frozen in liquid nitrogen. The same piece of tissue was dissected from the left cortices of sham and intact controls. Tissue was stored at -80°C until processed using TRIzol isolation method (Invitrogen, Grand Island, NY, USA), according to the manufacturer’s instructions. A slight modification was made regarding protein resuspension part of the protocol. Due to poor solubility of the protein pellet, it was grinded in liquid nitrogen and then resuspended in 1% SDS. The resulting supernatant was centrifuged at 10 000 g for 10 minutes and the supernatant was collected. Protein content was determined by the method of Markwell et al (32), and samples were kept at -20°C until use.

### RNA isolation and gene expression analyzes

After total RNA isolation and treatment with DNase (Fermentas, St. Leon-Rot, Germany), it was reverse transcribed to cDNA using the High-Capacity cDNA Reverse Transcription Kit (Applied Biosystems, Carlsbad, CA, USA). Quantitative real-time PCR (RTQ-PCR) was conducted using SYBR Green technology (Applied Biosystems, Carlsbad, CA, USA) and analyzed on AbiPrism 7000 (Applied Biosystems). The list of all primers (Invitrogen) designed in the free-access internet program Primer 3 is given in Table 1.

**Table 1:** List of primers used for real-time polymerase chain reaction.

Primer	Sequence 5′→3′	Length (bp)
β-Actin	f agattactgccctggctcct r acatctgctggaaggtggac	119
SOD2	f cagatcatgcagctgcacca r tcagtgcaggctgaagagca	133

### Western blot analysis

Immunoblot analysis was performed by standard protocol. Briefly, equal amounts of cortical tissue preparation (10µg/lane) were resolved by 7.5% SDS-PAGE gel according to Laemmli (33) and transferred to a polyvinylidene fluoride (PVDF) support membrane. Membranes were blocked in 5% bovine serum albumin (BSA) and incubated with rabbit polyclonal anti-SOD2 antibody (1:1000; Abcam, Cambridge, UK) overnight at 4°C. After incubation with donkey anti-rabbit IgG horseradish peroxidase conjugated secondary antibody (1:5000 dilution; Santa Cruz Biotechnology, Santa Cruz, CA, USA), a visualization was performed on x-ray films (Kodak) with the use of chemiluminescence. For each SOD2 blot, β-tubulin (Invitrogen) was used as a loading control. Optical densities of SOD2 immunoreactive bands were calculated as arbitrary units in the Image Quant program after local background subtraction. The results are expressed as the ratio of SOD2 and β-tubulin optical densities relative to the value obtained for intact control. Data presented in graphs are mean values ± standard error of the mean obtained from six immunoblots.

### Immunohistochemistry

After overnight fixation in 4% paraformaldehyde in 0.1 M phosphate buffer, pH 7.4, brains (3 per group) were cryoprotected in graded sucrose (10%-30% in 0.2 M phosphate buffer, pH 7.4) at 4°C. Brains were frozen in 2-methyl butane and stored at -80°C until cryosectioning. Immunohistochemistry was performed on 25 µm-thick coronal sections. Heated citrate buffer (pH 6) and 0.3% triton-X were used as antigen retrieval and membrane permeabilization steps, respectively. Blocking was done using 1% BSA. Colocalization of SOD2 with marker of neuronal cell bodies, NeuN, was examined with double immunofluorescence labeling using rabbit polyclonal anti-SOD2 (1:500, rabbit IgG, Abcam, Cambridge, MA, USA) and mouse monoclonal anti-NeuN antibody (1:500, Millipore, Vienna, Austria). Colocalization of SOD2 with marker of astrocytes was examined with double immunofluorescence labeling using rabbit polyclonal anti-SOD2 and mouse monoclonal anti-GFAP antibody (1:400, Abcam). Visualization of reaction was obtained using appropriate secondary fluorescent antibodies (1:250, Alexa Fluor 555 goat anti-mouse IgG and Alexa Fluor 488 donkey anti-rabbit, Invitrogen), and nuclei were marked using DAPI (Invitrogen). The sections were mounted in Mowiol (Calbiochem, San Diego, CA, USA). To test the specificity of the reaction, brain sections were treated in the same way with the omission of the primary antibodies. The sections were examined and photographed with Carl Zeiss Axiovert microscope (Zeiss, Gottingen, Germany).

Total fluorescence measurements were performed with Image J, as described in Burgess et al (34).‎ Total fluorescence intensity of SOD2 positive cells, labeled with NeuN or GFAP (respectively) was measured in 4 fields under the area of the screen ‎‎(0.38 mm2) in 4 sections per animal (n = 3) in the region around the injury (or corresponding ‎region in non-injured animals).

### Fluoro-Jade B staining and image analysis

Fluoro-Jade B staining was performed in order to visualize neuronal cells undergoing ‎degeneration and cell death. Brains and sections were prepared as for immunohistochemistry ‎procedure. The slides were first immersed in a basic alcohol solution consisting of 1% NaOH in ‎‎80% ethanol and distilled water, and incubated in 0.06% KMnO4 solution for 10 minutes. The slides ‎were transferred for 10 minutes to a 0.0001% solution of Fluoro-Jade^®^B (FJB, Chemicon International, ‎Temecula, CA, USA) dissolved in 0.1% acetic acid and then rinsed by three changes of distilled ‎water for 1 minute per change. The nuclei were marked using DAPI, and the slides were coverslipped with Mowiol (Calbiochem). The ‎sections were examined with Carl Zeiss Axiovert microscope (Zeiss). Cells ‎labeled with Fluoro-Jade B were observed as individual green spots. The number of degenerating ‎neurons labeled by FJB staining was counted in 3 fields under the area of the screen ‎‎(0.38 mm2) in 4 sections per animal (n = 3) in the region around the injury (or corresponding ‎region in non-injured animals). ‎

### Statistical analysis

All data are presented as mean ± standard error of the mean. Statistical significance of differences between the groups was determined using the one-way analysis of variance (ANOVA for repeated measures), with the treatment protocol and time post-surgery as factors. *P* value less than 0.05 (*P* < 0.05) or 0.001 (*P* < 0.001) was considered as significant.

## Results

**Figure 1:**
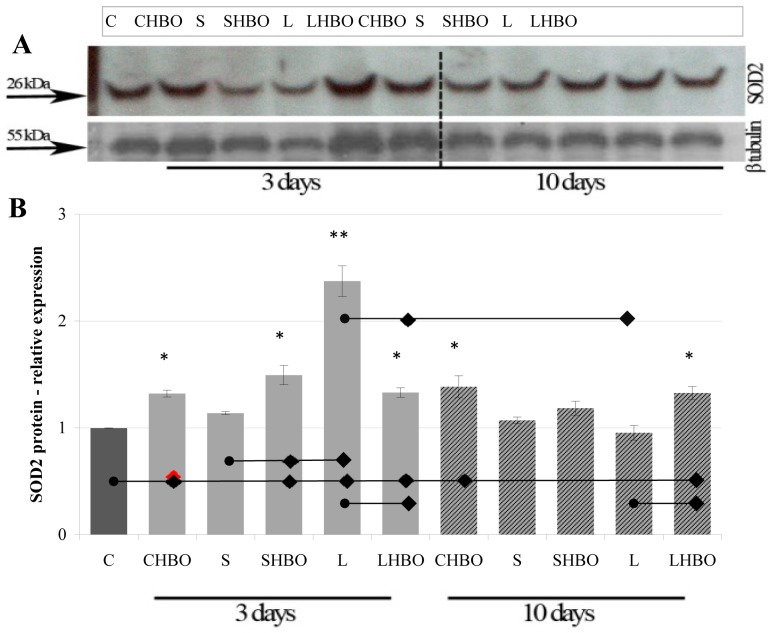
The effect of cortical stab injury (CSI) and hyperbaric oxygen therapy (HBOT) on superoxide dismutase 2 (SOD2) protein expression. The expression of SOD2 protein was assessed by Western blot analysis. Detection of β-tubulin was also carried out to confirm the equal loading of proteins. C – intact rats, CHBO – intact rats subjected to HBOT for 3 and 10 days, S – animals that underwent sham surgery, sacrificed at 3 or 10 dpi, SHBO – sham animals subjected to HBOT for 3 or 10 days, L – the animals that passed CSI sacrificed at 3 or 10 dpi, and LHBO – CSI rats subjected to HBOT for 3 or 10 days. (**A)** Representative immunoblots for SOD2 and β-tubulin. (**B)** Relative optical densities for SOD2 immunoreactive bands from six independent experiments were calculated as arbitrary units in the Image Quant program, and presented relative to control as mean ± standard error of the mean. The values of *P < 0.05 or **P < 0.001 were considered significant, this significance is shown as a dot (referent group) compared to a diamond (the group that is being compared).

Western blot analysis showed that the antibody used for SOD2 detection recognized one band at approximately 26 kDa. Quantification of total levels of SOD2 protein in all examined groups (Figure 1) revealed that, compared to control (0.997 ± 0.007), sham operation insignificantly altered SOD2 protein levels at both time points (3 and 10 dpi), while after CSI significant transient increase was observed. Thus, a significant 2.5 fold up-regulation of SOD2 protein levels detected at 3 dpi (2.373 ± 0.368), (*P* < 0.001, Figure 1) was followed by a reduction toward control levels 10 dpi (0.953 ± 0.137). The exposure to HBO for 3 and 10 days caused a significant (Figure 1) up-regulation of SOD2 in control and sham-operated groups. The exception was SHBO group, which was treated with HBO for 10 days and in which this increase was not significant. Interestingly, 3 days of HBO treatment considerably down-regulated SOD2 protein levels in the injured cortex (1.330 ± 0.088), when compared to untreated L group (2.373 ± 0.279), (*P* < 0.05), while after 10 days of HBOT there was a significant (39%, *P* < 0.05) up-regulation of SOD2 (1.327 ± 0.121), when compared to untreated L group (0.953 ± 0.137).

**Figure 2:**
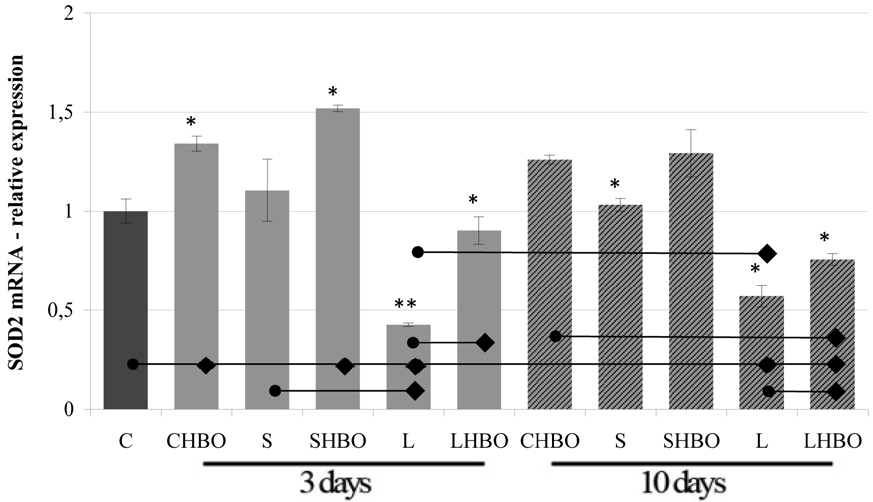
The effect of SCI and HBOT on SOD2 mRNA expression. Expression of SOD2 mRNA was measured by real time polymerase chain reaction (RTQ-PCR). The levels of each mRNA were normalized to those of the house-keeping gene β-actin. SOD2 mRNA relative expression values were compared to the control group and presented as mean ± standard error of the mean. The values of *P < 0.05 or **P < 0.001 were considered significant, this significance is shown as a dot (referent group) compared to a diamond (the group that is being compared). Acronyms are explained in the legend to Figure 1.

Surprisingly, RTQ-PCR analysis revealed that CSI significantly reduced SOD2 mRNA levels at 3 dpi (42.62 ± 1.74) (for 57%) and 10 dpi (57.06 ± 10.70), (for 43%) (*P* < 0.001 and *P* < 0.05 respectively, Figure 2) when compared to control (100 ± 12.09), while sham operation had no influence on SOD2 expression. However, although we noticed that HBOT significantly (*P* < 0.05) increased mRNA levels for SOD2 at both time points (2.1-fold at 3 dpi [90.11 ± 13.44] and 32% at 10 dpi [57.06 ± 10.70]) compared to the corresponding L group, they were still lower in comparison to control. Furthermore, it should be noted that the exposure to HBOT for 3 and 10 days increased mRNA levels for SOD2 in control (134.05 ± 7.59 after 3 days of HBOT and 125.99 ± 4.38 after 10 days of HBOT) and sham groups (151.84 ± 3.24 after 3 days of HBOT and 129.22 ± 23.56 after 10 days of HBOT), (*P* < 0.05, Figure 2).

**Figure 3:**
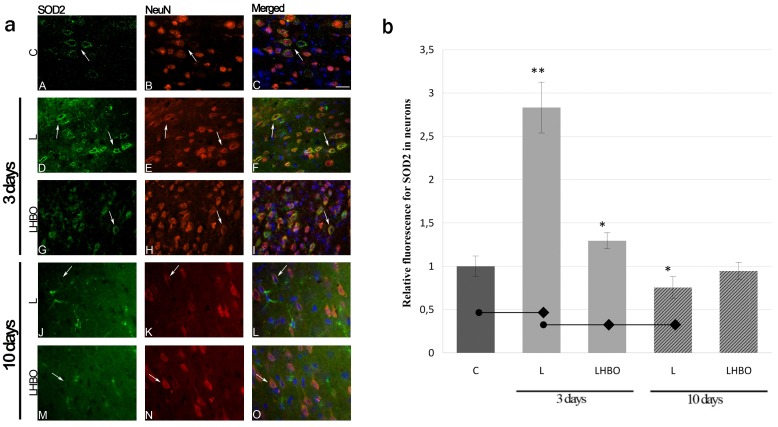
(**a**) The effect of CSI and HBOT on neuronal localization of SOD2. Double immunofluorescence staining with SOD2 (green) and NeuN (red) was used to detect neuronal localization of SOD2. Nuclei were stained with DAPI (blue). (**A, B, C**) In control, non-injured cortex expression of SOD2 was mainly seen in neurons (arrow). (**D, E, F**) CSI increased intensity of SOD2 staining in neurons at 3 dpi (arrows). (**G, H, I**‎) SOD2 staining was decreased to almost control levels after 3 days of repetitive HBOT. (**J, K, L**) Ten days after the CSI, SOD2 staining observed in neurons was similar to those seen in control sections. (**M, N, O**‎) SOD2 staining remained unchanged after 10 days of repetitive HBOT in comparison to 10dpi group. Scale bar applies to all images: 20 μm. (**b**) Quantification of fluorescence signal of SOD2 (green) in NeuN positive cells. Relative fluorescence intensity for SOD2 protein in neurons shows significant increase in SOD2 signal in neurons at 3 dpi, while after HBOT signal decreases to almost control levels at the same time point. Ten days after the CSI and after HBOT of the same group, total fluorescence intensity for SOD2 was unchanged compared to the control. All values were compared to the control group and presented as mean ± standard error of the mean. The values of *P < 0.05or **P < 0.001 were considered significant, this significance is shown as a dot (referent group) compared to a diamond (the group that is being compared).**‎** Acronyms are explained in the legend to Figure 1.

Double immunofluorescence staining was performed to detect the effect of CSI and HBOT on cellular localization of SOD2. Neuronal characterization of SOD2 was performed using NeuN as a marker of neurons and DAPI for the labeling of the cell nuclei. In the control sections, weak SOD2 immunoreactivity was mainly located in the neuronal soma. Three days after the injury, intense staining was observed in a number of neurons in the vicinity of the lesion site. Repetitive HBOT reduced SOD2 staining in the lesioned cortex when compared to injury alone. Ten days after the injury, the lesioned cortex of both L and LHBO groups showed down-regulation of SOD2 immunoreactivity, and only a paucity of SOD2^+^/NeuN^+^ neurons was seen. Quantitative analysis of fluorescence signal in neurons used to determine changes in SOD2 expression pattern revealed that at 3 dpi SOD2 fluorescence signal increased in NeuN^+^ cells about 3-fold around the lesion site (2.83 ± 0.76, *P* < 0.001), while exposure to HBO returned signal intensity to almost control levels (1.29 ± 0.18). Ten days after the CSI, the fluorescent signal in neurons in the perilesioned cortex was significantly decreased (0.75 ± 0.24) compared to 3 dpi and HBOT did not change this (Figure 3).

**Figure 4:**
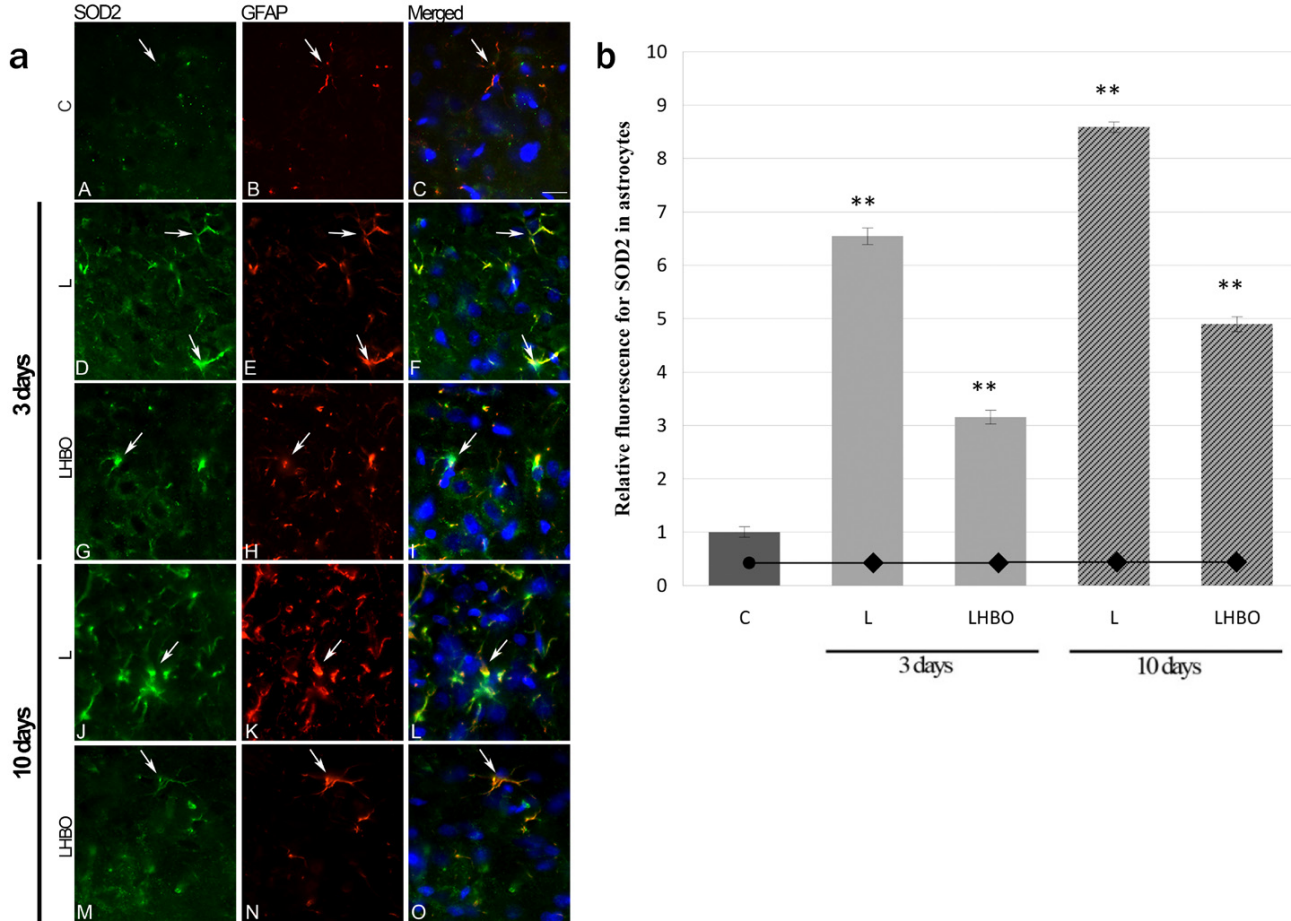
(**a**) The effect of CSI and HBOT on astrocytic localization of SOD2. Double immunofluorescence **‎**staining with SOD2 (green) and GFAP (red) was used to detect localization of SOD2 in astrocytes. **‎**Nuclei were stained with DAPI (blue). (**A, B, C**) In control, non-injured cortex low expression **‎**of SOD2 was seen in astrocytes (arrow). (**D, E, F**) CSI increased intensity of SOD2 staining in astrocytes at 3 dpi **‎‎**(arrows). (**G, H, I**) After exposure to HBOT, SOD2 staining in astrocytes was decreased compared to 3 dpi.**‎ (J, K, L**‎) Ten days after CSI, significant increase in SOD2 was observed in reactive astrocytes. (**M, N, O‎**) SOD2 staining in astrocytes was decreased after 10 days of **‎**repetitive HBOT compared to 10 dpi.**‎** Scale bar applies to all images: 20μm. (**b**) Quantification of fluorescence signal of SOD2 (green) in GFAP positive cells. Relative fluorescence intensity for SOD2 protein in astrocytes shows significant increase in SOD2 signal in astrocytes at 3 dpi, while after HBOT signal decreases by a factor of 2. Ten days after CSI about 4 times greater fluorescence signal was observed in glial fibrilary acidic protein (GFAP) positive cells compared to control. After 10 dpi with HBOT, SOD2 fluorescence signal was reduced by a factor of 2 as well. All values were compared to the control group and presented as mean ± standard error of the mean. The values of *P < 0.05 or **P < 0.001 were considered significant, this significance is shown as a dot (referent group) compared to a diamond (the group that is being compared). Acronyms are explained in the legend to Figure 1.

Interestingly, the decrease in neuronal SOD2 was accompanied by a significant up-regulation in astroglial cells, which was confirmed with double immunofluorescence staining for SOD2 and GFAP as a marker of astrocytes, while DAPI was used for the labeling of the cell nuclei. In control sections, scattered, weak SOD2 immunoreactivity was seen in astrocytes. Three days post-injury, SOD2 staining in reactive astrocytes surrounding the site of injury was obviously increased. Conversely, in LHBO group SOD2 immunoreactivity in GFAP-expressing astrocytes was lower. At 10 days after CSI, a huge number of GFAP-expressing hypertrophic astrocytes expressing SOD2 was seen in the close vicinity to the lesion site, while after 10 successive HBOTs their number was reduced. Quantitative analysis of astrocytes co-expressing SOD2 showed that, compared to control (1 ± 0.25), at 3 dpi intensity of SOD2 fluorescence increased 6.5-fold (6.54 ± 0.40), while fluorescence signal was reduced by a factor of 2 in the LHBO group (3.15 ± 0.33) (*P* < 0.001) (Figure 4). This relationship between SOD2 fluorescence signal in L and LHBO was maintained at 10 dpi (8.6-fold (8.59 ± 0.25) vs 4.9-fold [4.90 ± 0.36], respectively).

**Figure 5:**
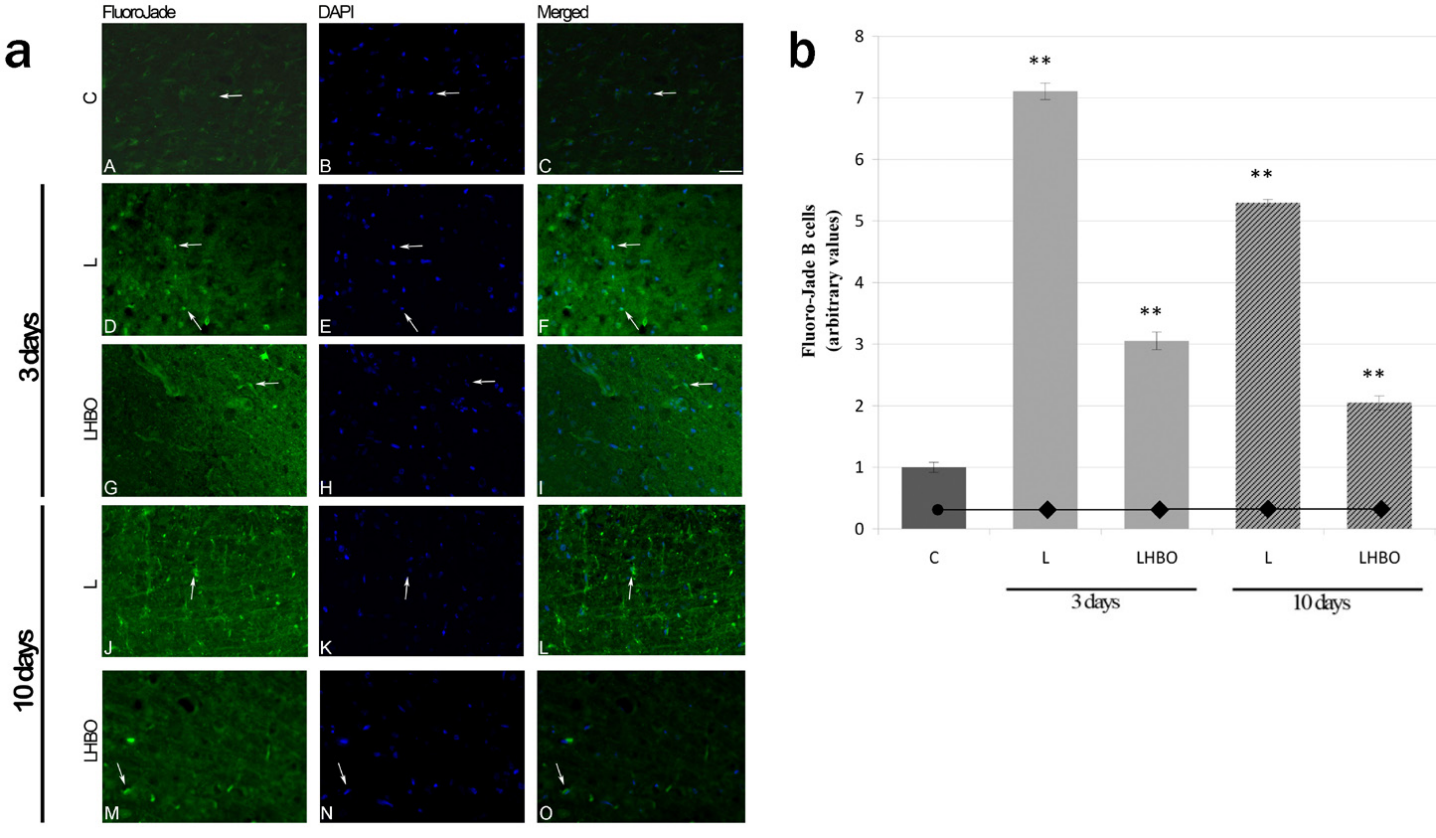
(**a**) Fluoro-Jade^®^B (FJB) staining of rat brains three and ten days after CSI and HBOT. Double immunofluorescence staining with FJB (green) and DAPI (blue) was used to detect ‎degenerating neurons. (**A, B, C**) Only a few neurons were FJB-positive in C group. (**D, E, F**) An increase in the number of FJB-positive cells was apparent 3 dpi. (**G, H, I**) Decrease in number of degenerated neurons after HBOT. (**J, K, L**) The number of FJB-positive cells is increased at 10 dpi. (**M, N, O**) After 10 HBOT, a decrease in number of degenerated neurons was seen. Degenerating neurons are marked with arrows. Scale bar applies to all images: 20 μm. (**b**) Quantitive analysis of Fluoro-Jade^®^B (FJB) staining of rat brains three and ten days after CSI and HBOT. All values were compared to the C group and presented as mean ± standard error of the mean. The values of *P < 0.05 or **P < 0.001 were considered significant, this significance is shown as a dot (referent group) compared to a diamond (the group that is being compared). Acronyms are explained in the legend to Figure 1.

FJB staining was performed to evaluate the effect of CSI and HBOT on neuronal degeneration. After CSI, the number of FJB-positive cells in the injured cortex increased at 3 dpi (7.11 ± 0.35) and 10 dpi (5.29 ± 0.14) 7- and 5.3-fold, respectively. The exposure to HBO for 3 and 10 days reduced the number of FJB-positive cells about 2.5-fold (3.05 ± 0.37 and 2.05 ± 0.30, respectively) in the injured cortex (Figure 5). In addition, no significant difference in the number of degenerating neurons was observed between the sections from C, CHBO, S, and SHBO groups (data not shown).

## Discussion

It is well known that ROS generated from the mitochondria have been implicated in different models of brain injury and that they may lead to apoptosis as a part of the secondary injury (12,35-37). Since SOD2 can be induced by various insults in the CNS, and is the first line of defense ‎against ROS, in this study we investigated the impact of CSI and HBOT on temporal pattern of its protein and gene expression. We found that CSI significantly altered SOD2 expression in a time-dependent manner, which is in agreement with a large body of evidence that is showing changes in enzyme expression and function ‎after brain insult (8,38-40).

We showed that in control brain sections, the majority of SOD2 immunofluorescence was observed in neurons, which is to be expected if we consider neurons as the brain cells with the highest oxygen consumption, constantly submitted to oxidative stress (41). It is important to note that after CSI remarkable, transient increase in SOD2 on the protein ‎level was detected at 3 dpi. At the cellular level, we demonstrated that enhanced SOD2 staining seen in neurons was even more pronounced in reactive astrocytes bordering the lesion site, indicating that observed SOD2 induction was mostly due to up-regulation in astrocytes. Enhanced ‎staining for SOD2 in neurons and astrocytes 48 hours after cortical injury in rats was previously ‎reported by Bidmon et al (8), who suggested that early increase in ‎SOD2 protects the cells in injured regions from superoxide-induced damage. Accordingly, increased protein levels of SOD2 that we detected at 3 dpi are most likely the response ‎to elevated ROS after the injury. Ten days after the CSI, SOD2 protein levels and fluorescence intensity in neurons returned to the control levels, which is also in agreement with the study by Bidmon et al (8). Similarly, in the model of excitotoxically induced ‎neurodegeneration, 2-fold increase in SOD2 protein observed at 24 hours after injection of quinolinic acid declined slowly over the following 10 days (42). Further, our result is consistent with earlier findings ‎that prolonged hypoxia/ischemia that occurs in injured tissue results in a general ‎decrease in SOD2 levels in neurons, and is discussed as an additional factor in causing ‎neuronal degeneration ‎(43). However, the decrease in neuronal SOD2 that we have noticed at 10 dpi was accompanied by significant up-regulation in highly reactive astroglial cells. A comparable extensive induction in reactive astrocytes and in the astroglial scar was showed for Cu/Zn SOD at 3, 5, and 7 days following excitotoxic injury in the postnatal rat brain (44). Consequently, astrocytes seem to be the main cell type that increases the total antioxidant capabilities in the nervous tissue after a lesion. The fact that they are more capable of handling oxidative stress conditions can explain their elevated resistance to cell death after an injury (44). In this regard, previous studies showed that Cu/Zn SOD-overexpressing astrocytes had increased resistance to oxidative damage (45). Having in mind that glial cells have an important role in supporting neighboring neurons, the appearance of injury-induced up-regulation of SOD2 in astrocytes seen at 3 and 10 dpi, therefore, seems to be a critical determinant for neuronal survival. Our results are in accordance with the study by Xu et al (46), who suggested that SOD2 overexpression in hippocampal astrocytes would improve the survival of CA1 neurons after transient forebrain ischemia.

Unexpectedly, our data point to discrepancies in SOD2 mRNA and protein levels. Namely, 3 days after the injury a significant increase in SOD2 observed at protein level was associated with ‎decreased SOD2 mRNA levels when compared to control. To our knowledge, we are the first to report a difference in SOD2 expression at protein vs ‎mRNA level after TBI. A difference between SOD2 ‎expression at protein and mRNA level was previously seen in cerebral endothelial ‎cells 6 hours following sublethal oxidative stress (47). Many studies were ‎performed in order to compare mRNA and protein expression on a large scale, and it ‎was indicated that mRNA levels only partly correlated with the corresponding protein ‎concentrations (48-50). The reason for this divergence could be ‎post-transcriptional regulation processes involving mRNA stability, translation initiation, ‎and protein stability. This dissimilarity between SOD2 mRNA and protein expression ‎emphasize the importance of investigation of posttranscriptional regulatory ‎mechanisms that can be unveiled only through integrated analyses of both proteins ‎and mRNAs.‎

Oxygen is one of the frequently used therapeutic ‎agents. Inattentive use of ‎oxygen at high partial pressures (pO_2_) can cause damage ‎due to excessive production and accumulation ‎of ROS (51). The degree of toxicity is shown to be dependent on treatment parameters: pressure and duration of the treatment (14,18,25,27,52). This‎ led to skepticism regarding the use of HBOT ‎‎(53), but also to numerous research studies trying to determine the optimal parameters for ‎HBOT in regard to both pressure of oxygen and duration of treatment. The clinically approved maximum pressure and duration of HBO exposure are 3 ATA and 120 minutes, respectively (31), although the most commonly used protocol for standard therapeutic purposes is slightly lower (1.8-2.8 ATA for 60-90 minutes) (53). In agreement with this knowledge, and according to our previous experience (19), we exposed rats to HBO. Using such HBOT protocol we avoided the potential risk of oxygen toxicity and prevented the appearance of convulsions (54,55). We started the HBOT protocol up to 2 hours after the surgery, having in mind the observations of other authors that efficacy of HBOT was considerably attenuated by 12 hours after TBI (18). The correctness of applied HBOT protocol and detection of possible histopathological changes after CSI and HBOT were evaluated using FJB staining, which is a relatively simple method for reliable detecting of dying neurons, regardless of the cell death cause (56), and comparable with results obtained using TUNEL staining, as another marker for neuronal death (57). Regarding HBOT effect on uninjured control groups, no significant difference in the number of FJB-positive cells was observed. Importantly, exposure to HBOT for 3 and 10 days significantly decreased the number of dying neurons, indicating that HBOT did not contribute to neuronal death after the brain injury. Neuroprotective effect of HBOT in brain injury has been demonstrated by several authors and may be mediated by preservation of mitochondrial membrane properties (58)

Our data showed that HBOT, when applied on non-injured ‎animals, increased SOD2 expression both on protein and mRNA ‎level, probably due to hyperoxic ‎challenge. Indeed, SOD2 is known as an inducible enzyme that may be activated in a variety of stressful conditions, including the changes in oxygen level (59). Furthermore, our results are in agreement with reports that HBOT increases SOD activity in several tissues (60). However, HBO is therapeutic modality with dual effects: it produces oxidative stress by itself but reduces oxidative stress when used in pathologic conditions (61). Similarly, we found that the exposure to HBOT for 3 and 10 days after the cortical injury reduced oxidative stress by returning SOD2 expression toward levels close to physiological. These results are consistent with our recent findings that activity of SOD, which was reduced after ablation of sensorimotor cortex, after exposure to HBO is elevated up to the values obtained in the control group (unpublished data). Protective effects of HBOT related to increased production of SOD2 have been demonstrated in the model of ischemia. It has been shown that HBO preconditioning improved ‎the tolerance to ischemia ‎‎(62). Moreover, when administered during ischemia, HBOT ‎reduced formation of hydroxyl radicals (63). It was proposed that the ‎underlying mechanism could be reduced glutamate cytotoxicity, owing to HBO-‎improved energy metabolism.

A significant amount of work was done in order to describe protective ‎effects of HBO preconditioning in different models of brain injury, and it was accepted that ‎initial oxidative stress acts as a trigger that up-regulates the antioxidant enzymes ‎including SOD2 (64). We have also recently reported that HBOT can improve locomotor performances in rats after cortical ablation (19).

In conclusion, the results of this study imply that application of HBOT, via altering SOD2 ‎expression, may attenuate imbalance between oxidants and antioxidants that occurs after brain ‎injury, and in that way contributes to the maintenance of pro-/antioxidant homeostasis. ‎Moreover, a reduced number of dying neurons in the injured cortex suggested that HBOT could ‏contribute to reducing secondary injury after TBI‎. Although precise mechanisms ‎remain elusive, we believe that presented results may be of ‎importance when considering HBO as ‎an adjunctive therapy for patients with brain injury.‎‏ ‏Further studies should be performed to ‎elucidate whether observed changes in SOD2 expression at protein and mRNA level are ‎accompanied with corresponding changes in SOD2 activity and ROS status.‎

##
